# Life-threatening pleural hemorrhage following intrapleural enzyme therapy and successful treatment with fibrin-thrombin sealant pleurodesis: a case report

**DOI:** 10.1186/s13256-015-0775-5

**Published:** 2015-12-18

**Authors:** Simon V. Vun, David G. Lance

**Affiliations:** 1 Cardiac and Thoracic Surgical Unit, Flinders University, Sturt Road, Bedford Park, SA 5042 Australia; 2 Cardiac and Thoracic Surgical Unit, Flinders Medical Centre, Flinders Drive, Bedford Park, SA 5042 Australia

## Abstract

**Introduction:**

Intrapleural fibrinolytic enzyme therapy is a potentially surgery-sparing treatment for poorly resolving parapneumonic effusion and empyema. It is safe in the majority of patients, however the most significant risk associated with this treatment is severe bleeding secondary to pleural hemorrhage. Contraindications for intrapleural enzyme therapy are not widely agreed upon and little is known about how to treat this difficult and potentially lethal hemorrhagic complication.

**Case presentation:**

An independent 82-year-old Caucasian man presented to hospital with an empyema complicating community-acquired pneumonia and coincidental pulmonary embolus. He was initially commenced on intravenous antibiotics, pleural drainage and anticoagulation, however failed to improve significantly and was commenced on intrapleural fibrinolytic enzyme therapy. Shortly after, he suffered severe pleural hemorrhage that was uncontrollable despite emergency thoracotomy and washout. Subsequent hemostasis was achieved after re-exploration and application of topical fibrin-thrombin sealant spray. The patient survived and was discharged home.

**Conclusions:**

Intrapleural enzyme therapy can be effective in loculated parapneumonic effusion and empyema, but massive pleural hemorrhage can complicate its use. Pleural hemorrhage appears to be associated with anticoagulation or coagulopathy, and can be difficult to manage. This case adds to the body of data on bleeding complications following intrapleural enzyme therapy, and to the best of our knowledge is the first report of fibrin-thrombin sealant use in this setting.

## Introduction

Pleural infection is common and is increasing globally. Unfortunately, patients experience high morbidity and mortality nearing 20 % [1]. Conventional treatment strategies consist of appropriate antibiotics and drainage of the pleural space with tube thoracostomy. However, pleural collections are often viscous and loculated by fibrinous septations, resulting in inadequate drainage and necessitating surgery [2].

The intrapleural administration of agents to improve drainage was first described using streptokinase and streptococcal deoxyribonuclease (DNase) nearly 65 years ago [3]. Since then, there have been studies showing varied results, and a number of randomized controlled trials (RCTs) have attempted to address this. The first Multicenter Intrapleural Sepsis Trial (MIST1), that randomized patients with parapneumonic effusions to intrapleural streptokinase or placebo, surprisingly failed to show any benefit in clinical outcomes such as length of stay, need for surgery or mortality [4]. The subsequent MIST2 trial attempted to assess the efficacy of alteplase alone or in combination with DNase, since DNase is thought to decrease effusion viscosity [5]. Combination treatment significantly improved radiological resolution, reduced frequency of surgical referral, and hospital length of stay compared to placebo. Overall serious adverse effects did not differ statistically significantly between treatment groups, however two patients who experienced intrapleural hemorrhage were both in the combination group.

Whether or not anticoagulation constitutes an absolute contraindication is not widely agreed upon. And although bleeding complications seem to be perceived as rare [6], there are a number of reports of severe pleural bleeding requiring transfusion or emergency surgery [7–9]. In those requiring surgery, an active focal bleeding site is infrequently found and thoracotomy and decortication is often described [7–9].

We present a case of a patient with life-threatening refractory pleural hemorrhage following the intrapleural administration of alteplase and DNase that was managed successfully by thoracotomy and adjunctive topical fibrin-thrombin sealant.

## Case presentation

**Fig. 1:**
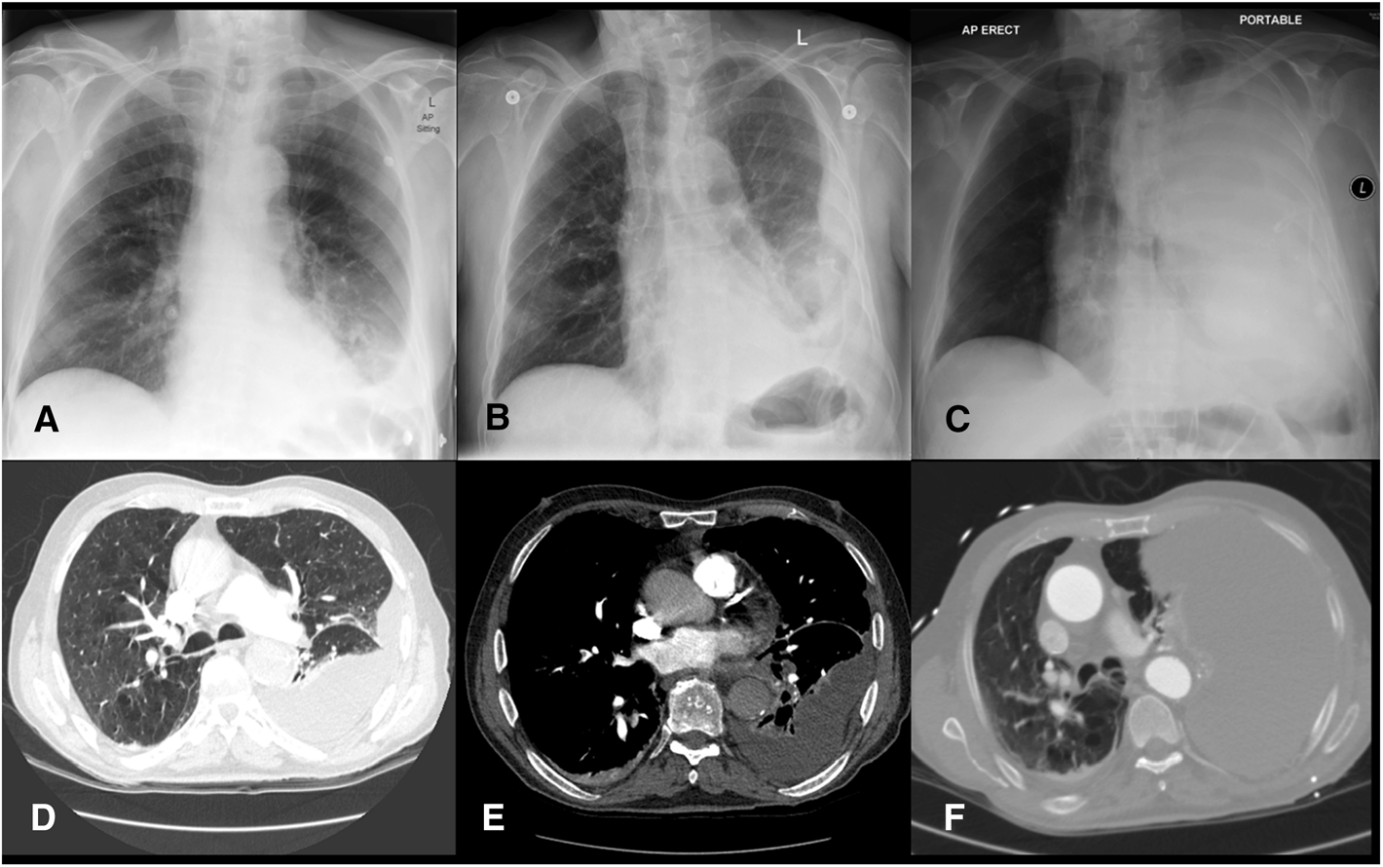
**a** Chest radiograph on presentation at hospital. **b** Chest radiograph after initial pigtail drain insertion. **c** Portable chest radiograph during medical emergency call. **d** Transverse contrast-enhanced computed tomography image on presentation to hospital – lung window. **e** Pulmonary embolus in division of right pulmonary artery. **f** Computed tomography angiogram following medical emergency team call

An 82-year-old Caucasian man was admitted to a local private hospital with a 6-day history of cough, malaise and left-sided pleuritic chest pain. A chest radiograph demonstrated left basal consolidation consistent with community-acquired pneumonia (Fig. 1a). Blood tests on admission are shown in Table 1. A computed tomography (CT) pulmonary angiogram demonstrated a right-sided pulmonary embolism, left lower lobe consolidation and moderate left-sided pleural effusion. (Fig. 1d-e). Intravenous piperacillin/tazobactam, subcutaneous enoxaparin (65 mg twice daily) was commenced, and a 6 French pigtail tube thoracostomy was performed under ultrasound guidance (Fig. 1b). Turbid yellow fluid was drained and results of the analysis are shown in Table 2. After 3 days, suboptimal clinical and radiologic resolution resulted in transfer to our institution under the respiratory physicians where intrapleural enzyme therapy was commenced with alteplase (10 mg in 50 mL of saline, 12-hourly) and dornase alfa (Roche AG, Basel, Switzerland)) (5 mg in 50 mL of saline 12-hourly).

**Table 1:** Admission blood tests

Hemoglobin	135 g/L
White cell count	10.3 × 10^12/L
Neutrophils	8.2 × 10^9/L
Platelets	318 × 10^9/L
C-reactive protein	132.9 mg/L
Sodium	130 mmol/L
Potassium	4.2 mmol/L
Urea	10.6 mmol/L
Creatinine	91 umol/L
Albumin	25 g/L
Total protein	68 g/L
Lactate dehydrogenase	258 U/L
D-dimer	2310 ng/mL
APTT	52 seconds
INR	1.2

*APTT* activated partial thromboplastin time, *INR* international normalized ratio

**Table 2:** Pleural fluid analysis

pH	7.8
Albumin	14 g/L
Total protein	42 g/L
Lactate dehydrogenase	3570 U/L
Glucose	<1.0 mmol/L
Cytology	Mesothelial cells, macrophages and mixed inflammatory cells. No atypical or malignant cells. No micro-organisms seen
	Acid-fast bacilli not detected
Culture	Light growth of *Staphylococcus aureus*

On the second day (after the third dose) of enzyme therapy, a medical emergency call was activated for sudden hypotension (blood pressure 70 mmHg systolic) and respiratory distress. Drainage had become sanguineous and then ceased, presumably due to obstruction of the small-bore drainage tube. An examination revealed hypovolemic shock and a chest X-ray demonstrated complete opacification of the left hemithorax with mediastinal deviation toward the right (Fig. 1c). His hemoglobin level dropped to 91 g/L, and our patient was transferred to the intensive care unit (ICU) and was transiently responsive to fluid boluses. An urgent CT angiogram demonstrated collapse of the left upper and lower lobes due to a massive pleural effusion, but no contrast extravasation to indicate active bleeding (Fig. 1f). A 32 French tube thoracostomy was performed in intensive care, resulting in the immediate drainage of over 3000 mL of sanguineous fluid. Our patient remained unstable and was transferred to the operating theater for left thoracotomy and exploration for bleeding.

A posterolateral thoracotomy was performed through the fifth intercostal space. On entering the pleural cavity, a further 3600 mL of blood was immediately evacuated with cell salvage. The pleural cavity was then explored systematically. Anteriorly, a small adhesion from the superior segment of the lower lobe appeared to have torn and was bleeding and was controlled with point diathermy. The lung appeared inflamed, hyperemic and a small abscess cavity was noted in the lower lobe, which was evacuated. The entirety of the pleural cavity was then examined directly and with the assistance of vidoeoscope. This revealed widespread hyperemic pleural surfaces, which bled on contact. No focal cause for massive hemorrhage could be found. Pleural biopsy would later show fibrous and fibrinous pleuritis thickened with abundant inflammatory granulation tissue. Following copious washout the incision was closed with two drains and our patient was returned to the ICU. A total of 2500 mL of cell-salvaged blood was returned to the patient.

Despite aggressive correction of coagulopathy, the drains accumulated over 1800 mL of blood over 3 hours, and our patient was returned to theater for repeat exploration and hemostasis. Again, only diffuse pleural hemorrhage was encountered and no discrete bleeding point was seen. The pleural cavity was packed extensively with gauze packs for 20 minutes in an attempt to gain control, but was not successful. The pleural cavity was then sprayed with fibrin-thrombin sealant (Tisseel, Baxter AG, Vienna, Austria), the lungs re-inflated, and the chest closed in an attempt to control bleeding via pleurodesis.

Our patient was returned to the ICU and required vasopressor support and ongoing transfusions despite minimal drain output. A moderate apical effusion developed on chest X-ray, but this was managed expectantly. Hepatic and renal dysfunction ensued requiring hemofiltration. Extubation was achieved on the fifth postoperative day and drains removed on the ninth after draining a total of 2760 mL. A further 14 days in the ICU were required for weaning of renal support and subsequently our patient was transferred to the ward. After a brief period of rehabilitation, our patient was discharged home. Table 3 shows total blood products administered.

**Table 3:** Blood products administered from date of surgery (units)

Day 0	Packed red blood cells	21
	Fresh frozen plasma	17
	Platelets	5
	Cryoprecipitate	15
	Recombinant factor VII	6.0 mg
	Albumin 4 %	1
Day 1	Packed red blood cells	3
	Fresh frozen plasma	4
	Platelets	1
Day 2	Packed red blood cells	2
	Fresh frozen plasma	4
	Platelets	2
Day 3	Packed red cells	1
Day 4	Packed red cells	2

**Table 4:** Summary of results of literature review

Study	Study type	Sample	Intrapleural therapy used	Anticoagulation or antiplatelet use	Hemorrhagic complications
Thommi *et al*. 2012 [11]	Single centre placebo controlled randomized controlled trial	68 adults	Placebo or alteplase	Unspecified	Hemorrhage requiring transfusion in two patients
Rahman *et al*. 2011 [5]	Multicentre placebo controlled randomized trial	210 adults	Placebo or alteplase or DNase or alteplase + DNase	Unspecified	Two intrapleural hemorrhages and one hemoptysis in alteplase + DNase group. Two episodes of gastrointestinal bleeding in the DNase group.
Maskell *et al*. 2005 [4]	Multi-centre placebo controlled randomized trial	427 adults	Placebo or streptokinase	Unspecified	Seven hemorrhages (local or systemic) in streptokinase group, six in placebo group
Nie *et al*. 2014 [10]	Meta-analysis of randomized trials	879 adults, 98 children	Placebo, streptokinase, urokinase, tPA	Unspecified	Nonsignificant increase in severe side effects
Piccolo *et al*. 2014 [13]	Retrospective observational study	107 adults	tPA and DNase	Unfractionated heparin × 1, chronic liver disease and ↑prothrombin time × 1	Hemorrhage requiring transfusion in two patients
Anevlavis *et al*. 2011 [7]	Case series	Two adults	Alteplase	Therapeutic dose tinzaparin (14,000 IU), Prophylactic tinzaparin (3500 IU)	Two massive pleural hemorrhages
Chai and Kuan, 2011 [17]	Case report	One adult	Streptokinase	Nil	Massive pleural hemorrhage
Goralski *et al*. 2009 [9]	Case report and literature review	One adult	Alteplase	Nil	Severe pleural hemorrhage
Gervais *et al*. 2008 [12]	Retrospective observational study	66 adults	Alteplase	Warfarin × 1, low-molecular-weight heparin (dalteparin 5000U TDS) × 2, unfractionated heparin × 1	Five major pleural hemorrhages in four patients who were anticoagulated. No hemorrhage in those receiving prophylactic anticoagulation or clopidogrel
Ruiz *et al*. 2006 [8]	Case report	One adult	Alteplase	Nil	Massive pleural hemorrhage

*DNase* deoxyribonuclease, *tPA* tissue plasminogen activator

## Discussion

Despite first being performed over 65 years ago, understanding of the use of intrapleural therapy continues to evolve. A recent meta-analysis of ten randomized controlled trials concluded that compared to placebo, intrapleural fibrinolytic therapy decreased the chance of needing surgical intervention and length of hospital stay, but lead to a nonsignificant increase in severe side effects [10]. In severely ill patients who pose high surgical risk, intrapleural therapy may mitigate the need for thoracic surgery. A particularly high-risk situation exists where one-lung ventilation is complicated by ipsilateral pulmonary emboli, as was present in this case. However, contraindications to intrapleural enzyme therapy, in particular regard to therapeutic anticoagulation, are not widely agreed upon. In MIST 1 and 2, anticoagulation did not constitute exclusion criteria [4, 5], and in the recent RCT by Thommi *et al*. patients were still eligible too as long as international normalized ratio (INR) was <4, partial thromboplastin time <100 s, platelets >60,000 [11]. Their most recent recommendation is that the INR and partial thromboplastin time be <4 and <50 s, respectively [6]. However, there have been a number of cases reporting major hemorrhage in patients who are coagulopathic or anticoagulated. Gervais *et al*. reported a series of 66 patients who received intrapleural tissue plasminogen activator (tPA), of which there were five major pleural hemorrhages in four patients, all of whom were therapeutically anticoagulated at the time [12]. Notably, of those who did not bleed, 38 were receiving prophylactic subcutaneous heparin or dalteparin, 12 were receiving aspirin, and two were receiving clopidogrel. Piccolo *et al*. described a series of 107 patients treated with intrapleural tPA/DNase in which two patients experienced nonfatal hemorrhage requiring blood transfusion [13]. One was coagulopathic from chronic liver disease, the other dialysis dependent and anticoagulated for concurrent pulmonary embolism. The latter patient died 19 days later due to sepsis. In addition, there have been a number of individual case reports of pleural hemorrhage, causing hemorrhagic shock, following intrapleural enzyme therapy. Ruiz *et al*. reported massive hemothorax, following intrapleural alteplase for complicated parapneumonic effusion in a 31-year-old woman [8]. The patient was not reported as being coagulopathic or anticoagulated at the time, was resuscitated, and underwent thoracotomy. No source of bleeding was seen and it could not be attributed to traumatic drain insertion. A further two case reports of massive hemothorax, resulting in hypovolemic shock, were reported by Anevlavis *et al*. [7] in adults who received intrapleural alteplase while receiving low-molecular-weight heparin (tinzaparin). Additionally, Goralski *et al*. report a 40-year-old man with human immunodeficiency virus (HIV) on antiretrovirals and end-stage renal failure who received intrapleural alteplase for a parapneumonic effusion, suffered a cardiac arrest due to hemorrhagic shock [9]. He was resuscitated, underwent an emergency thoracotomy for evacuation of hemothorax. No bleeding source was identified and bleeding continued until administration of platelets, fresh frozen plasma, cryoprecipitate, and activated factor VII, returned his coagulation profile to baseline. Table 4 summarizes the findings of the literature review. The above cases indicate that serious bleeding complications may be more common than they are perceived, and a recent review of intrapleural enzyme therapy called for further documentation of complications and their management [14].

Our case is similar to those discussed above, in that the patient developed massive hemothorax in the setting of therapeutic anticoagulation, however different in that he received combination alteplase with DNase intrapleurally. The contribution of DNase to bleeding is uncertain. This case report adds to the body of data on severe pleural hemorrhage following intrapleural enzyme therapy in patients who are anticoagulated, raising the awareness of this complication among clinicians. In such patients, it is recommended that the risks and benefits of intrapleural therapy versus surgery should be carefully evaluated in a multidisciplinary team environment. Moreover, those who receive intrapleural enzymes in the setting of bleeding risk factors should be closely monitored. This report is also unique given that hemorrhage continued despite thoracotomy, aggressive correction of coagulopathy, and hemostasis was achieved only after the application of topical fibrin-thrombin sealant, Tisseel. Fibrin-thrombin sealants were first used during hemostasis by Bergal in 1909 [15]. Modern fibrin-thrombin sealants, which consist of human origin fibrinogen and thrombin, and antifibrinolytic, aprotinin, have been approved by the FDA for hemostasis, adhesion and sealing. In thoracic surgery, they have been successfully employed to treat persistent bronchopleural fistulas following lung resection or recurrent pneumothorax [16]. Although not a new method of hemostasis per se, this is the first case report to describe the use of fibrin-thrombin sealant to successfully treat refractory pleural hemorrhage following intrapleural fibrinolytic enzyme therapy and anticoagulation. For junior surgeons, including trainees, who have not yet come across severe refractory pleural hemorrhage in practice, we believe this case report to offer valuable reference of the successful use of topical fibrin-sealant in this context.

## Conclusions

Severe pleural bleeding may complicate the use of intrapleural enzyme therapy for complicated parapneumonic effusion or empyema, particularly in anticoagulated or coagulopathic patients. In severe refractory pleural hemorrhage, the use of topical fibrin-thrombin sealant can be an effective life-saving adjunct to surgery.

## Consent

Written informed consent was obtained from the patient for the publication of this case report and any accompanying images. A copy of the written consent is available for review by the Editor-in-Chief of this journal.

## Abbreviations

APTT: Activated partial thromboplastin time
CRP: C-reactive protein
CT: Computed tomography
DNase: Deoxyribonuclease
HIV: human immunodeficiency virus
ICU: Intensive care unit
INR: International normalized ratio
RCT: Randomized controlled trial
tPA: Tissue plasminogen activator
